# Supplementary material for: Machine Learning Analysis of Hyperspectral Images of Damaged Wheat Kernels
Source: Sensors (Basel). 2023 Mar 28;23(7):3523. doi: 10.3390/s23073523 (PMC10098892; doi:10.3390/s23073523)
Supplement: Supplementary file 1 [file sensors-23-03523-s001.zip › Figure S1.pptx]

## Slide 1
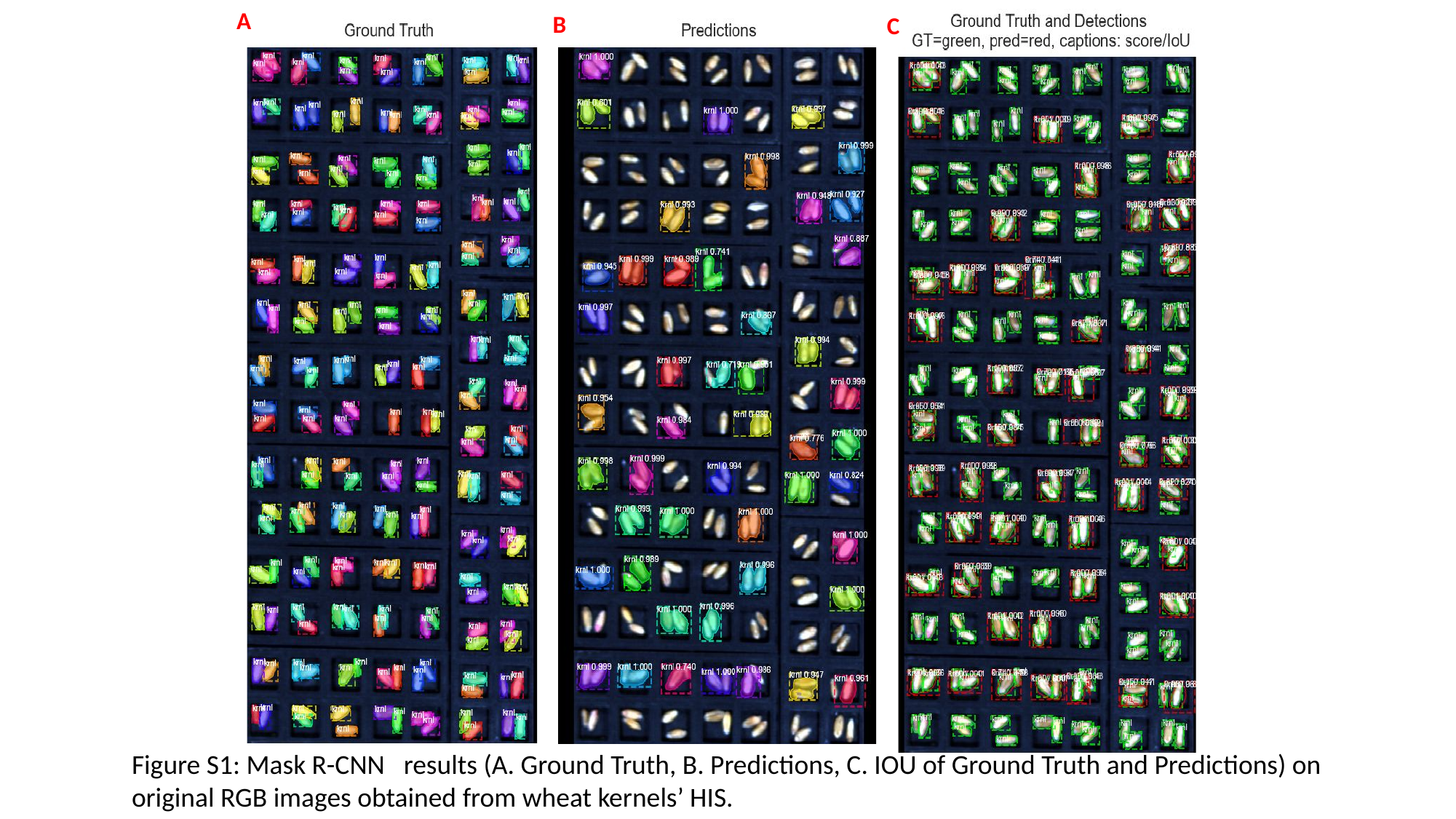

A
B
C
Figure S1: Mask R-CNN   results (A. Ground Truth, B. Predictions, C. IOU of Ground Truth and Predictions) on original RGB images obtained from wheat kernels’ HIS.
