# Supplementary material for: Machine Learning Analysis of Hyperspectral Images of Damaged Wheat Kernels
Source: Sensors (Basel). 2023 Mar 28;23(7):3523. doi: 10.3390/s23073523 (PMC10098892; doi:10.3390/s23073523)
Supplement: Supplementary file 1 [file sensors-23-03523-s001.zip › Figure S3.pptx]

## Slide 1
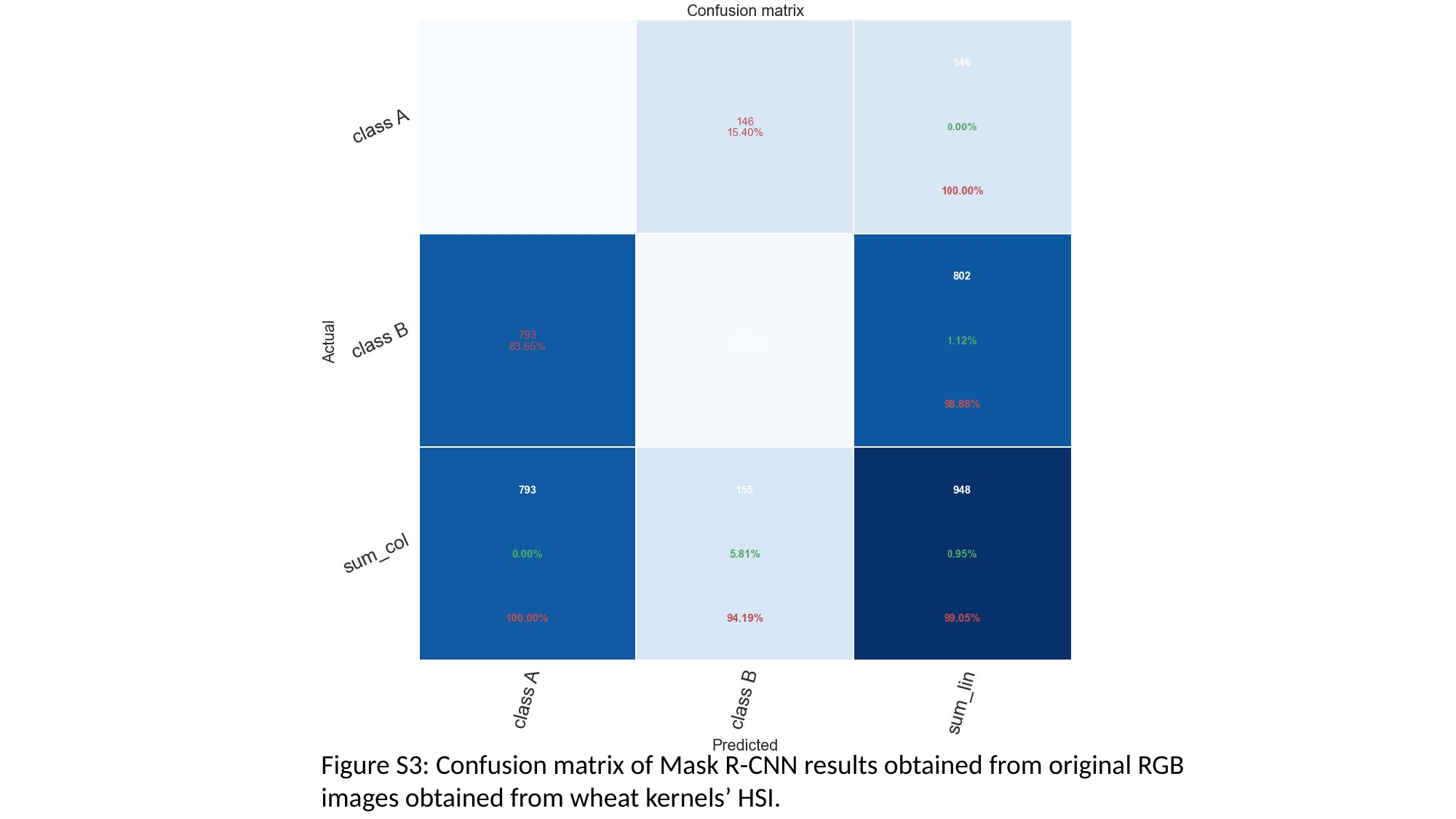

Figure S3: Confusion matrix of Mask R-CNN results obtained from original RGB images obtained from wheat kernels’ HSI.
