# Supplementary material for: Machine Learning Analysis of Hyperspectral Images of Damaged Wheat Kernels
Source: Sensors (Basel). 2023 Mar 28;23(7):3523. doi: 10.3390/s23073523 (PMC10098892; doi:10.3390/s23073523)
Supplement: Supplementary file 1 [file sensors-23-03523-s001.zip › Figure S6.pptx]

## Slide 1
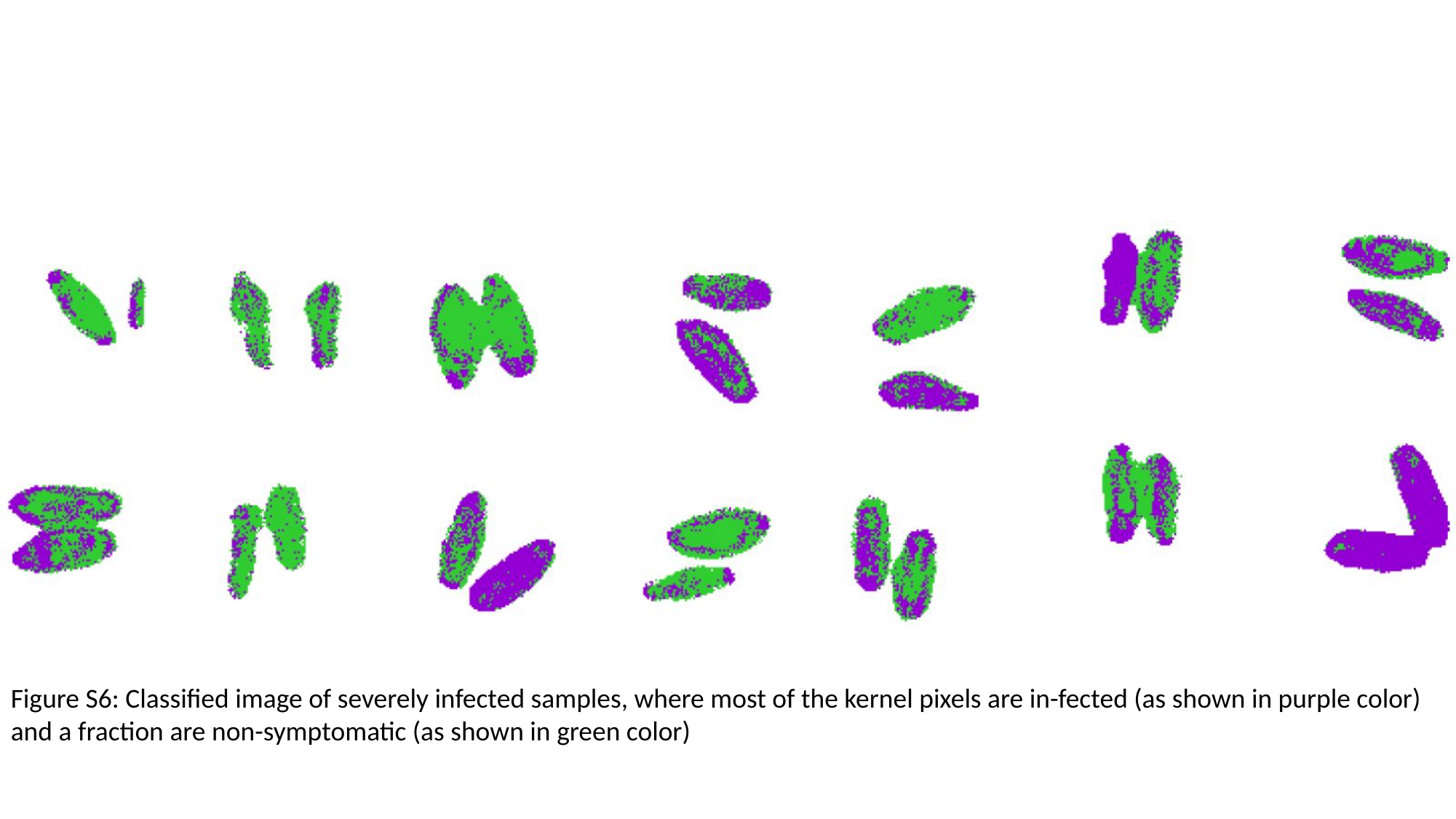

Figure S6: Classified image of severely infected samples, where most of the kernel pixels are in-fected (as shown in purple color) and a fraction are non-symptomatic (as shown in green color)
