# Supplementary material for: Machine Learning Analysis of Hyperspectral Images of Damaged Wheat Kernels
Source: Sensors (Basel). 2023 Mar 28;23(7):3523. doi: 10.3390/s23073523 (PMC10098892; doi:10.3390/s23073523)
Supplement: Supplementary file 1 [file sensors-23-03523-s001.zip › Figure S7.pptx]

## Slide 1
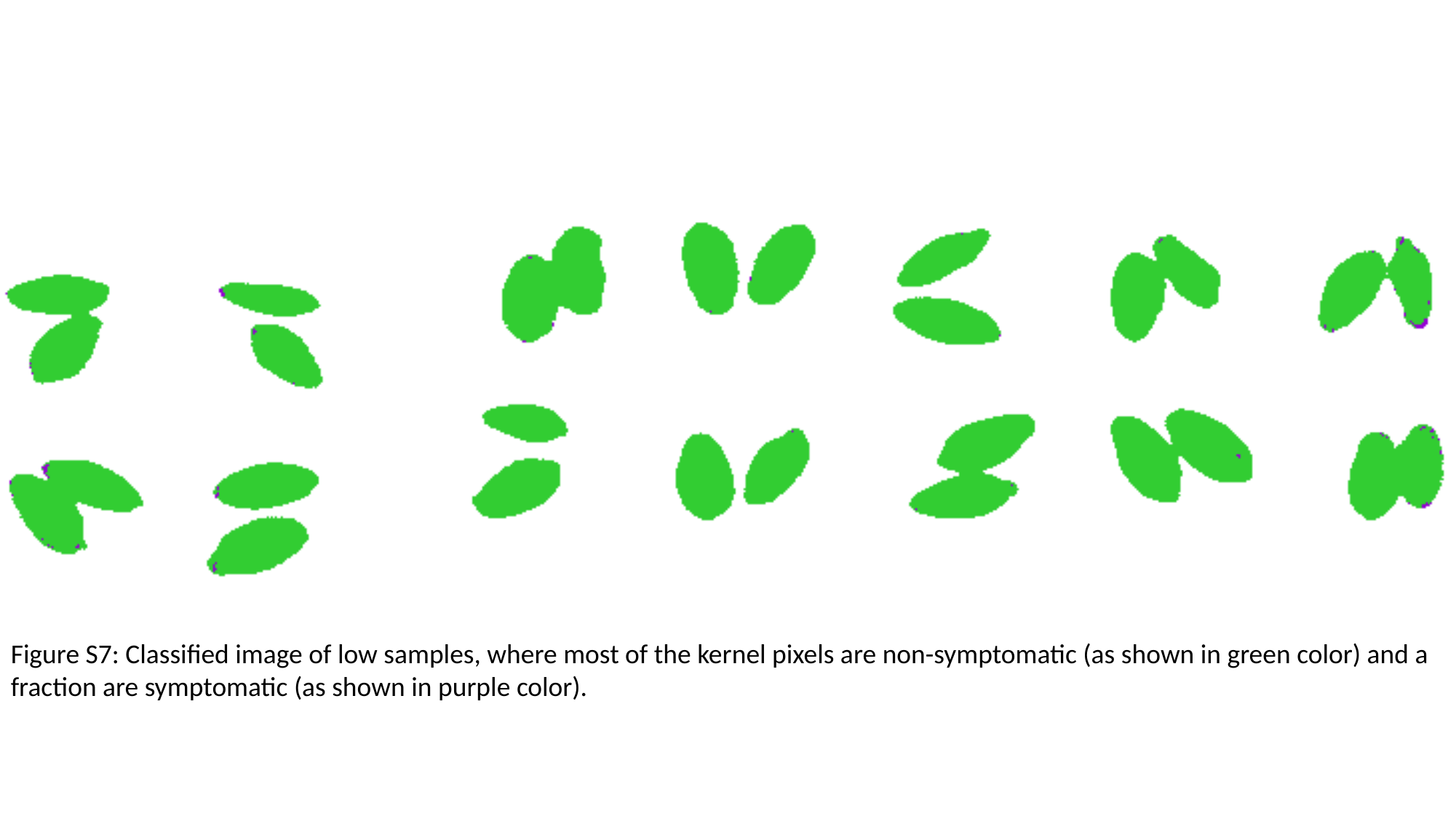

Figure S7: Classified image of low samples, where most of the kernel pixels are non-symptomatic (as shown in green color) and a fraction are symptomatic (as shown in purple color).
