# Supplementary figures and images for: Machine Learning Analysis of Hyperspectral Images of Damaged Wheat Kernels
Source: Sensors (Basel). 2023 Mar 28;23(7):3523. doi: 10.3390/s23073523 (PMC10098892; doi:10.3390/s23073523)

## Slide 1
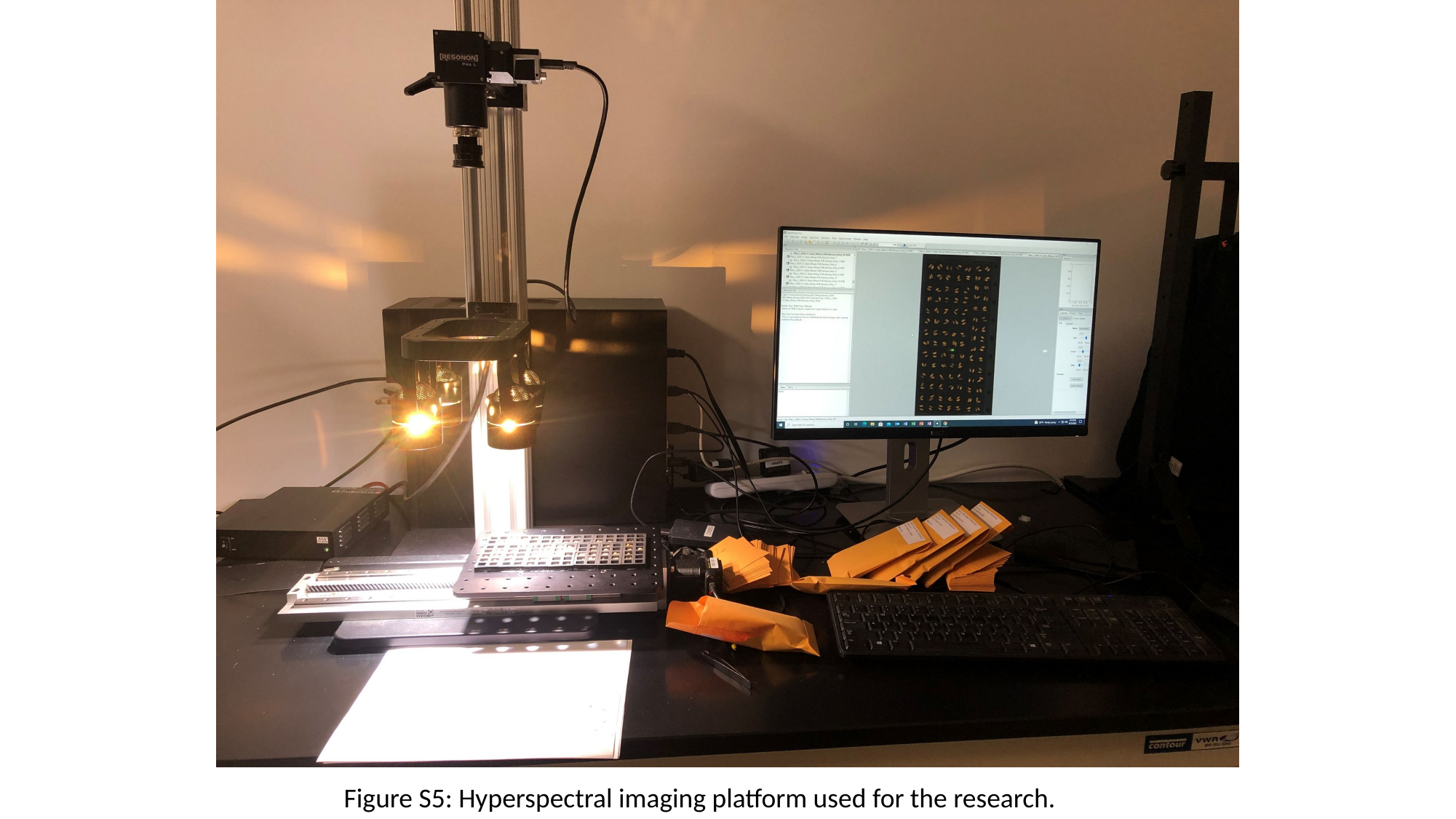

Figure S5: Hyperspectral imaging platform used for the research.

Supplement: Supplementary file 1 [file sensors-23-03523-s001.zip › Figure S5.pptx]
